# Towards mHealth applications for pet animal owners: a comprehensive literature review of requirements

**DOI:** 10.1186/s12917-025-04658-3

**Published:** 2025-03-21

**Authors:** Laura Haase, Brita Sedlmayr, Martin Sedlmayr, Dagmar Monett, Julia Winter

**Affiliations:** 1 Institute for Medical Informatics and Biometry, Carl Gustav Carus Faculty of Medicine, Technische Universität Dresden, Dresden, Germany; 2 Department of Cooperative Studies – Computer Science, Berlin School of Economics and Law, Alt-Friedrichsfelde 60, 10315 Berlin, Germany

**Keywords:** mHealth, Veterinary medicine, Requirements gathering, Medical laypersons, Animal owners, Literature review, Decision support system

## Abstract

**Background:**

Veterinarians experience high workloads and stress levels in their daily work, of which they need to be relieved as much as possible. The general public is showing great interest in digital health services. At the same time, animal owners and veterinarians are seeing telehealth services as particularly positive for triage aspects in veterinary medicine. One approach to support veterinarians may be to enable pet owners to, for instance, make informed decisions on how urgent their animal needs to be examined by a veterinary professional through an mHealth application. For this, stakeholder requirements need to be gathered, which should provide as a starting point for the development of such a decision support system.

**Results:**

955 publications were screened, resulting in the extraction of 10 requirements to mHealth applications for animal owners from 13 publications. Most frequently mentioned aspects were: ensuring complete information input by the user (6 mentions) and displaying a disclaimer about application limitations prominently (5 mentions).

**Conclusions:**

Most of the extracted requirements focus on the design of the human-computer interface, revealing this as a crucial point to such applications, especially in guiding animal owners through information and ensuring understanding, particularly of application limitations. However, the small number of included publications shows that primary research in this field, in general, and in this specific topic in particular, is needed in order to fully reflect the requirements for an mHealth application to help animal owners decide on their animal’s need to be examined by a veterinary professional.

**Supplementary Information:**

The online version contains supplementary material available at 10.1186/s12917-025-04658-3.

## Background

Nowadays, veterinarians experience high levels of stress in their work life due to, among other issues, a lack of skilled veterinary personnel compared to the number of animals needing veterinary care [1, 2]. Meanwhile, the general public is showing great interest in digital health services [3], and veterinarians as well as animal owners perceive the support of triage in veterinary medicine through telehealth services as particularly positive [4, 5].

Mobile Health (mHealth) apps are defined as mobile applications that “aim to promote and maintain health by supporting behavior change and/or decision making” [6]. While long-term studies often do not exist, short-term evaluations of the impact and effectiveness of mHealth applications in human medicine show beneficial effects for patients for different areas, e.g. reducing hospitalization for asthma patients, improving pulmonary disease symptoms or reducing death and hospitalization from heart failures [7]. In a review of mHealth applications for diabetes mellitus patients, improved bloodwork, as well as improved self-efficacy and self-care were found as a result of the app use [8]. Symptom checkers like the Ada Health app present themselves as close to the efficiency of general practitioners in evaluating urgency and suggesting diagnoses [9]. Since a big part of veterinary experts’ everyday life consists of advising specifically pet animal owners regarding their animals’ current health status and doing triage via phone [10], we suggest that an application to support the animal owners in determining their pet’s need to be examined by a veterinary expert may help reducing the veterinarians workload. As animal owners may need this kind of support in different locations, which may also be limited in internet access [11], and since most people do own a smartphone [12] and nearly one quarter of those already did use health-related apps in 2023 [13], this research is focused towards requirements to mHealth applications.

Yet, research regarding decision support systems in veterinary medicine is barely available [14]. Compared to many human medicine applications, veterinary medicine presents the special challenge of animals not being able to express their own symptoms verbally, leading to the anamnesis being completely third-party-based [15, 16]. In human medicine textbooks this is described as highly complex and prone to errors for trained medical staff [17, 18]. Since animal, and especially pet owners might be veterinary laypersons that are emotionally involved in the situation and prone to biases due to their individual beliefs, health concepts, and intended effects on others (e.g. ensuring others see them as competent caregivers to their animal) [11], the task of determining the animal’s need for veterinary care is even more difficult to solve.

As a first step in the development of an application for supporting animal owners in the determination of their animal’s need to be examined by a veterinary expert, a literature search was conducted. The major goal was to find out about requirements to such mHealth applications that are specific to the user group of animal owners and their characteristics. In this paper, details on the literature review are presented together with its results.

## Methods

### Methodological approach

The paper focuses on the identification of stakeholder requirements. Those refer to aspects that are expected from an application and/or that must be considered when creating an application concept. They therefore form the basis for system requirements to be defined later on [19], which, however, are not in the scope of this paper.

When starting the research on requirements to mHealth applications for animal owners, an initial search for existing publications centered around this very topic was done. As no publications on this specific topic were found, an approach for a comprehensive literature review was designed. Given the lack of dedicated databases or journals specifically covering veterinary informatics [14], the review was structured in two phases:

1. A narrative literature review with broad search terms to gather an initial understanding of the topic and identify relevant keywords for a more focused search.
2. A systematic literature review based on the keywords identified in the first phase to ensure a structured and reproducible review process.

**Fig. 1:**
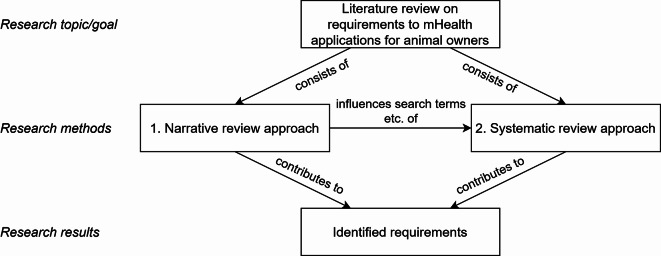
Methodological approach used in the literature review

The overall methodological approach is illustrated in Fig. 1.

### Narrative review

The narrative literature review approach was based on Google Scholar database [20], which was selected to draw from an extensive and interdisciplinary pool of publications. The terms “*veterinary telemedicine*,” “*veterinary telehealth*,” “*veterinary mHealth*,” and “*veterinary informatics*” were selected as umbrella search terms, each used in an individual search with the “Citations excluded” setting of the database. The final search was carried out on December 8th, 2022.

#### In-/Exclusion criteria

For the title and abstract screening of the search results, the following list of exclusion criteria was used:

(A) not written in German or English language;
(B) not a full text publication from a scientific journal or a scientific conference, a doctoral thesis, or a recommendation from a veterinary specialist group;
(C) focus on concrete system implementations or implementation technologies;
(D) focus on ethical, legal or financial aspects;
(E) focus on telehealth in the form of monitoring (continuous surveillance of the animal);
(F) focus on specific animal species/breeds;
(G) focus on specific veterinary medical specialties (e.g. oncology);
(H) focus on limited geographical areas (e.g. individual countries); and.
(I) focus on the perceptions and training of veterinary medicine students.

Criterion (A) was selected due to the reviewer’s language skills. Criterion (B) was added to ensure scientific and/or practical validity and relevance of the publications to be included. Criteria (C-I) were selected to focus the publications to be included on requirements independent of a specific environment and therefore valid for a wide variety of applications.

#### Screening process and data extraction

As, from the beginning, the narrative approach was set to be complemented by a systematic literature review, and its aim was primarily to identify relevant search keywords, there was no need for completeness in this review approach. Therefore, Google Scholar’s feature of sorting search results in accordance to the tool-identified relevance of publications was used for screening. Results were screened (with decreasing inclusion of publications) until all publications for at least 30 search results were excluded.

Publications included from the title/abstract screening were then exported into the research assistance tool Zotero [21] to help with duplicate identification and source management.

Afterwards, an attempt was made to obtain the full text of the remaining publications free of charge (through open access publishing or publisher deals of the authors’ universities). The publications’ full texts were then screened with regard to the exclusion criteria listed above and the following additional criterion:

(J) no ex- or implicitly stated requirements to mHealth applications for animal owners.

Implicitly stated requirements were defined as those that are made with regard to, for instance, telemedicine offers, but are transferrable to mHealth applications as well. Statements from which requirements may be derived (e.g. *veterinary medical competence of the pet owners is poor*/*veterinary medical vocabulary is often used incorrectly by animal owners*) were not classified as requirements.

All publications that were left in the review after the full text screening were then included for data extraction.

The literature references and mentioned telehealth guidelines from the included publications were similarly screened for further publications to be considered for the review.

### Systematic review

Based on keywords from publications included in the narrative review approach, complemented with terms related to those keywords and the review scope, the following search query was designed for the systematic literature review:

*(“mHealth” OR “mobile app” OR “smartphone application” OR “mobile health” OR “teletriage” OR “telehealth” OR “telemedicine” OR “informatics” OR “software development” OR “software design” OR “application design” OR “application development”)*.

*AND*

*(“veterinary” OR “animal” OR “pet” OR “dog” OR “cat” OR “horse” OR “ferret” OR “rabbit” OR “guinea pig” OR “rat” OR “mouse” OR “hamster” OR “hedgehog”)*.

*AND*

*(“owners” OR “caregivers” OR “laypersons” OR “guardian” OR “keeper”)*

The query was tested multiple times before coming up with the presented final version, and afterwards adapted to the search language of each of the databases (see Additional File 1) that were considered, namely Scopus, ACM Digital Library, IEEE Xplore and EBSCO Host (searched for title, abstract and keywords) and Web of Science, PubMed and CAB Direct (searched for title and abstract as those did not offer a specified keyword search). Those databases were selected due to their relation to computer science, agriculture, and human or veterinary medicine.

The final search was carried out on January 30th, 2023.

The search results were then imported into Zotero [21] to help with duplicate identification. An included conference proceeding was removed and instead the publications being part of this proceeding added to the list of publications for better assessment. Assisted by the systematic review assistance tool Rayyan [22], the title and abstract screening of the publications was carried out by two authors.

#### In-/Exclusion criteria

Publications were excluded from the review following the same exclusion criteria as for the narrative review approach. Only two changes were made this time: First, in the first criterion (A), both Spanish and French languages were no longer exclusion criteria as one of the authors would have been able to translate those publications if found. Second, an additional criterion for the screening was introduced:

(K) no strong focus on veterinary telemedicine, veterinary telehealth, veterinary informatics, veterinary mHealth, or veterinary teletriage.

This criterion was not needed in the narrative literature review as those keywords were already included in the initial search string and therefore were surely part of the review.

#### Screening process and data extraction

All publications included after the title/abstract screening were then tried to be obtained in full text, free of charge for the authors (through open access publishing or publisher deals of the authors’ universities). With all obtained publications, a full-text screening was conducted, again separately by two authors. Subsequently, literature references from the publications included so far were searched for further candidate publications for reviewing, based on their title.

After completion of the full-text screening, all included publications were carefully reviewed for all incorporated requirements for mHealth applications for pet owners. This was done independently by two authors who marked each explicitly or implicitly mentioned requirement (see definition of exclusion criterion (J)) in the manuscripts. Both authors then went through each manuscript in a joint meeting, compared their markings and, if only one author marked a passage, discussed that case in detail to reach a consensus. In the same joint meeting, parts of the text were then summarized and clusters of thematically similar aspects were formed, so that after several rounds of reformulation, the final list of requirements was created. The items on this list were then grouped according to their overall theme for a better overview.

## Results

### Narrative review

Following the methodology described above, 800 publication titles and abstracts were screened. After title/abstract screening and duplicate removal, 59 publications were sought for retrieval. 58 publications were available to the authors and therefore part of the full-text screening.

**Fig. 2:**
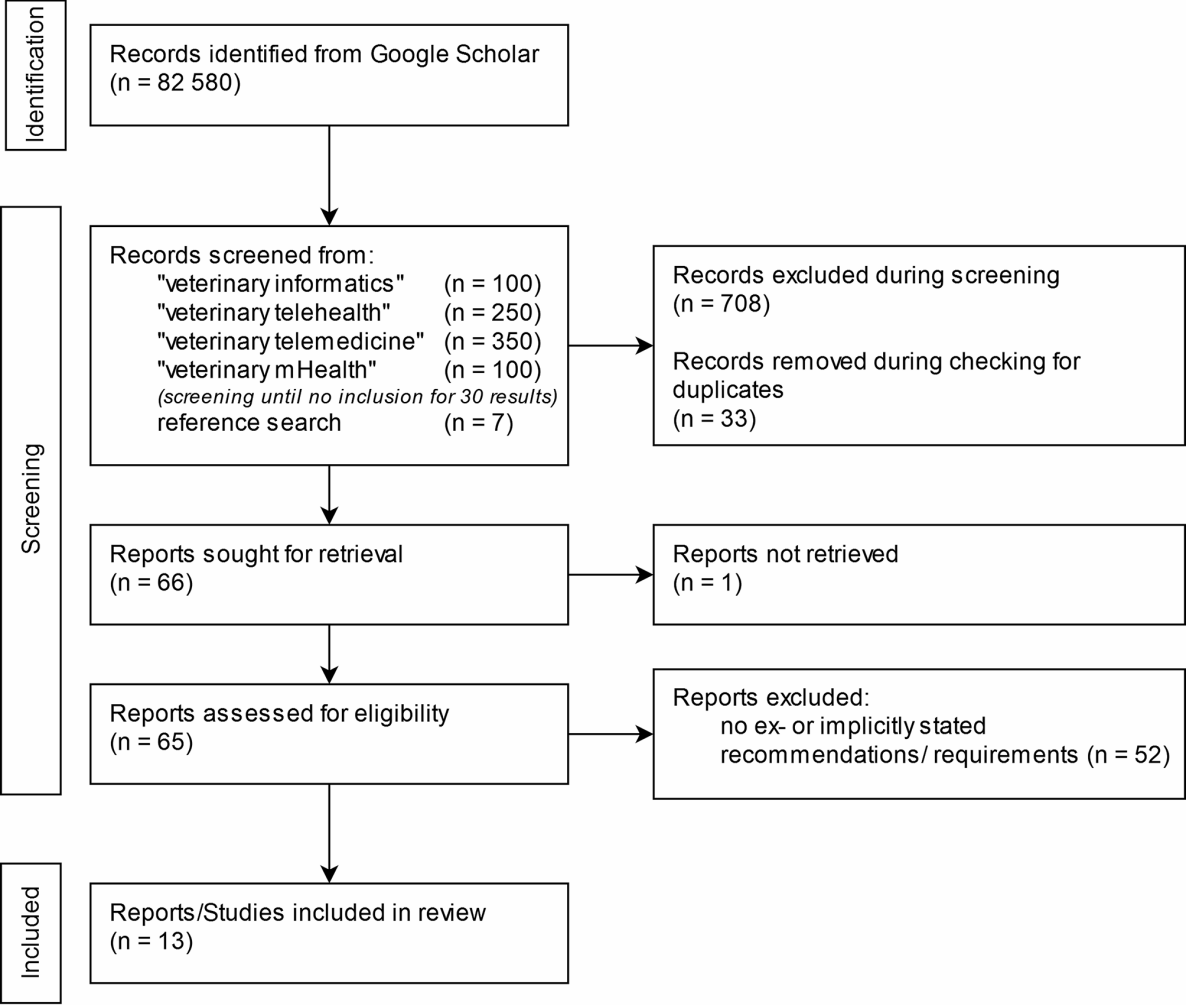
Preferred reporting items for systematic reviews and meta-analyses (PRISMA) chart of the narrative literature review

After the full-text screening, 9 publications were selected for data extraction. Additionally, from literature references and telehealth guidelines mentioned, another 4 publications were included, resulting in a total of 13 publications containing ex- or implicitly stated requirements to mHealth applications for animal owners (see Fig. 2).

A preliminary version of the results of the narrative review approach (without including the 2 most recent publications) was already published in [23].

### Systematic review

**Fig. 3:**
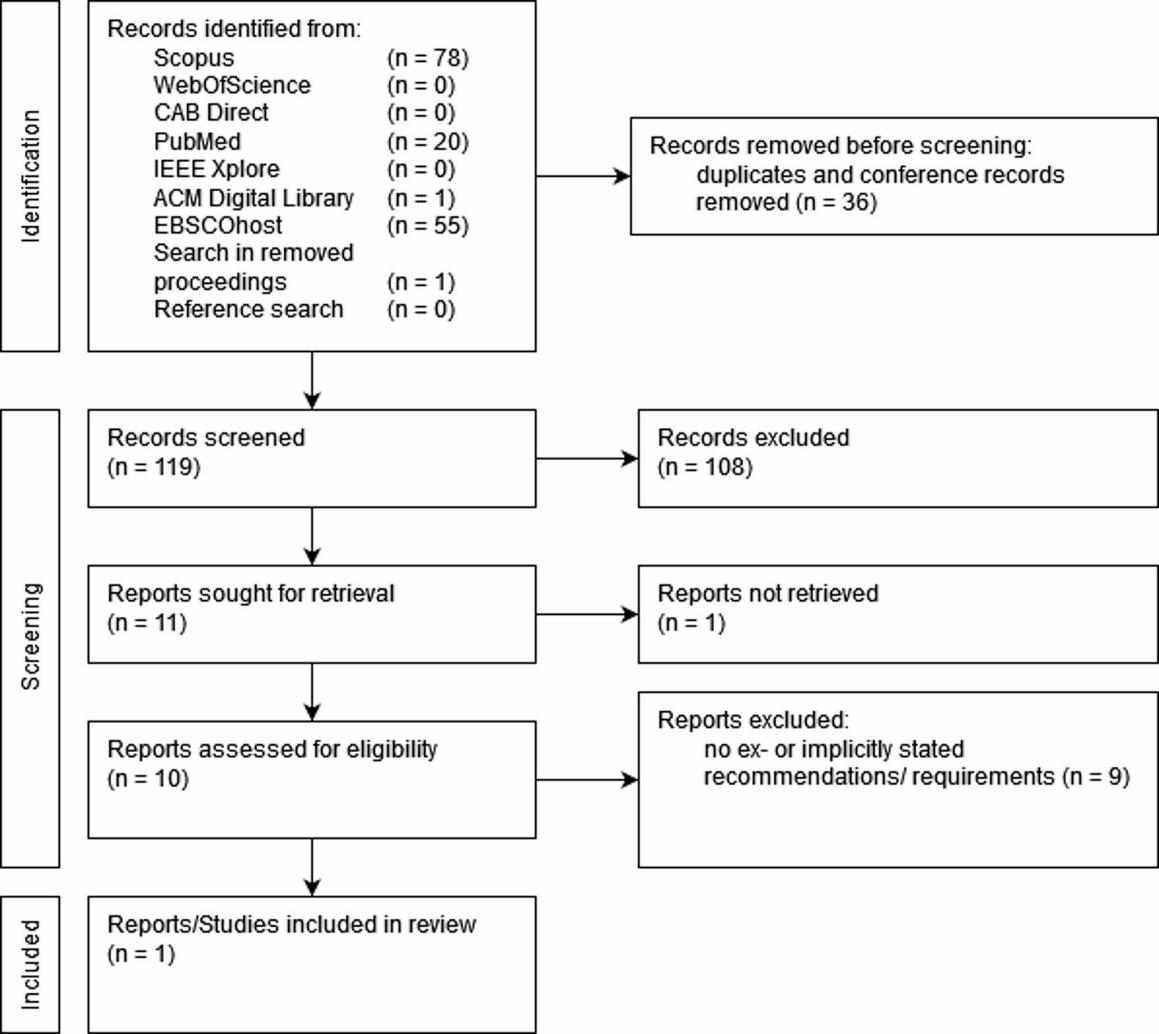
PRISMA chart of the systematic literature review

Following the methodology for the systematic review approach described above, 154 publications were identified after searching the literature databases. 36 duplicates and conference records were removed, leading to 119 publication titles and abstracts being screened. 11 publications were included for further evaluation in the full-text screening and sought for retrieval. From those, 10 were available to the authors, which were then screened in full text. As a result, 1 publication was included in the review. The screening of the titles of the literature references from the included publication let to no further inclusions (see Fig. 3).

### Joint review

**Fig. 4:**
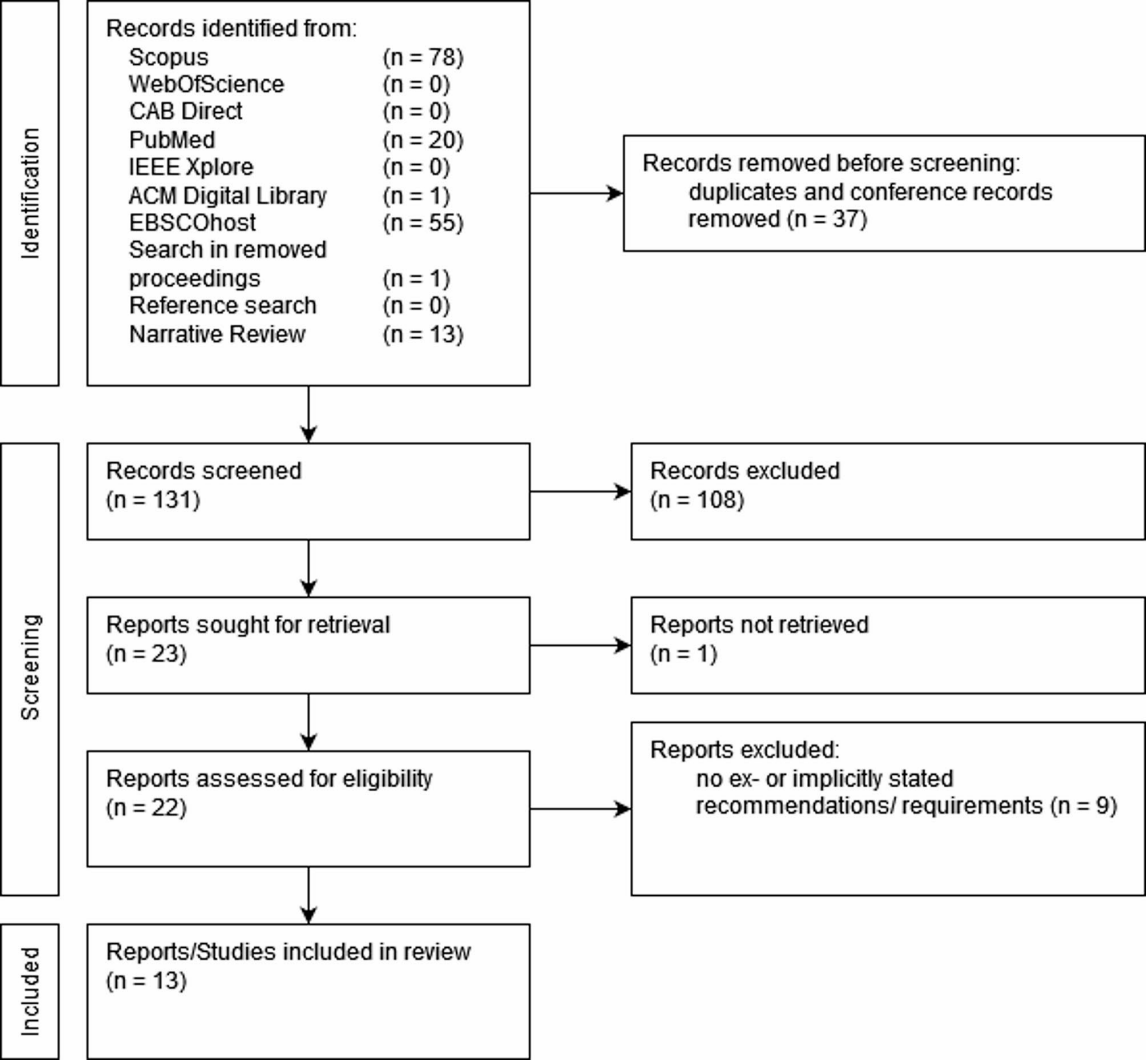
PRISMA chart of the joint literature review

The publication identified through the systematic review process was already included in the identified publications from the narrative search approach (see Fig. 4).

From the resulting 13 publications, 10 that stated requirements to mHealth applications for animal owners either implicitly or explicitly were identified and clustered into three categories, according to their content:

#### Human-computer interface design

(i) ensure complete information input by the user [14, 24–28];
(ii) display a disclaimer about application limitations prominently [26, 29–32];
(iii) make and display everything in a simple way to facilitate understanding [14, 27, 28];
(iv) display an (emergency) vet contact [26, 28, 31];
(v) use terminology adequate to the user’s clinical and mental models of diseases [33];
(vi) display a hint to talk about the application usage with the vet [31];
(vii) ensure the usage of the application to be in the user’s everyday routines to make it less likely to fail at critical times [27];

#### Application goals

(viii) set the support of the vet and/or their tasks as the overall application goal [34];

#### Functionalities

(ix) adapt the output depending on the specific animals’ case (e.g. species, breed, age, medical history, etc.) [14, 35]; and.
(x) document everything for the consulted veterinarian to prevent the animal owner from telling everything twice or thrice [28].

Requirements (i), (ii), (iii), (iv), (v), (vi) and (x) were identified by the authors as requirements to be highlighted due to their particular importance to mHealth applications for animal owners.

The results are mostly (9 out of 13) from the last five years before the search (since the beginning of 2017). The oldest publication is from 2001. No publications by the same authors or in the same publication medium were found. 6 publications come from journals, 3 are guidelines from specialist groups, 1 publication comes from a book, and 1 publication from a conference. The subject areas of the publication media are veterinary medicine (7), medical informatics (2), medical technology (1), and education (1).

## Discussion

### Principal results

A very small number of publications to be included was found in the review, although the review was very comprehensive due to the combination of the narrative and systematic strategies. This may indicate that there are few specific requirements for mHealth applications for animal owners in the literature. As expected, included publications are from various disciplines, with veterinary medicine being the most represented one. The variety of publishing disciplines also reflects in the small number of included publications from the systematic review approach, which shows the hurdles in spreading knowledge in the field of veterinary informatics.

The extracted requirements focus on the design of the user interface. In particular, instructions for the user to ensure complete input and understanding of the information available (for instance on the limitations of the application) are mentioned. Especially the use of adequate terminology and simple presentation is also advised from reviews of human medicine symptom checker/decision support applications [36, 37] and other mHealth related usability research (e.g [38–42]).

Strong user guidance through the user interface may be even more necessary than in similar applications (like mHealth applications for third-party anamnesis) in human medicine. This may be the case as most people develop a mental model of human diseases through personal, family, or similar experiences that they can fall back on when asked about their own symptoms or those of others. Yet, most of those models cannot be transferred to veterinary medicine [10, 43], which may lead to misjudgments or missing some of the animals’ symptoms. These challenges must be met by a suitable design of the human-computer interface, an essential conclusion of the research reported in this paper. How such a design may look like should be the center of further research.

### Limitations

A limitation of the review methodology is that the narrative review process was carried out with only one reviewer. Furthermore, the search terms of the systematic review were of high specificity, which may have led to few publications being excluded that were marked exclusively with terms that required a low level of specificity. In the query of the systematic review, farm animals and farmers were explicitly not included as search terms, because the farming profession requires more knowledge and experience than private animal owners need to have [11]. This might lead to different requirements to a veterinary mHealth application. The more general search terms “program”/“programme”/“system” were also not included in the final query, because a test run of the query showed the search results to increase by around 4 500% (over 7 000 publications to be examined) due to their low specificity (e.g. initially including topics as study programs).

Other possible biases in the obtained results might have been introduced by the specified languages or by searching using keywords in English. In addition, it cannot be guaranteed that all relevant publications have been found, since the presence of requirements in publications with a broader thematic focus (as described above) makes a specific search very difficult. Moreover, it cannot be assumed that all existing publications are indexed in the searched databases. Due to a lack of availability to the authors, no search could be carried out in other veterinary-related databases (e.g. Agricola, Embase and Biosis). This might also have led to the exclusion of relevant literature.

## Conclusions

The specified requirements for mHealth applications for pet owners were extracted from a limited number of publications. Due to this, the requirements identified can only serve as a base for further research. To expand them and be able to suggest possible, well-serving implementations, additional research should be carried out. This could be done, for instance, through identifying veterinary information processes currently used by animal owners. The design of the human-computer interface should be worked out iteratively, in a collaborative process together with animal owners as well as veterinary experts. This should be done to ensure both veterinary correctness of and adequacy to influential characteristics of the animal owners (like mental models and beliefs, knowledge, skills, cultural norms). In the development process, looking for transferable research results from third-party-based anamnesis in human medicine (e.g. in pediatrics) could be beneficial [44]. However, those aspects need to be treated with caution and checked comprehensively. Despite its limitations and possible research extensions, the literature review presented in this paper fulfills its initial goal and is a first step towards an mHealth application for animal owners to support them in determining their animal’s need to be examined by a veterinary expert. Finally, the results presented here show that the design of the human-computer interface is particularly important. Other already addressed aspects and concrete information to be presented in an mHealth application are essential as well.

## Electronic supplementary material

Below is the link to the electronic supplementary material.

Supplementary Material 1

## Data Availability

Data is provided within the manuscript or supplementary information files.

## Abbreviations

mHealth: Mobile Health
PRISMA: Preferred reporting items for systematic reviews and meta-analyses
