# Supplementary material for: Towards mHealth applications for pet animal owners: a comprehensive literature review of requirements
Source: BMC Vet Res. 2025 Mar 21;21:190. doi: 10.1186/s12917-025-04658-3 (PMC11927274; doi:10.1186/s12917-025-04658-3)
Supplement: Supplementary file 1 — Supplementary Material 1 [file 12917_2025_4658_MOESM1_ESM.docx]

## Additional File 1 - Database Search Strings

### Scopus

TITLE-ABS-KEY(("mHealth" OR "mobile app" OR "smartphone application" OR "mobile health" OR "teletriage" OR "telehealth" OR "telemedicine" OR "informatics" OR "software development" OR "software design" OR "application design" OR "application development") AND ("veterinary" OR "animal" OR "pet" OR "dog" OR "cat" OR "horse" OR "ferret" OR "rabbit" OR "guinea pig" OR "rat" OR "mouse" OR "hamster" OR "hedgehog") AND ("owners" OR "caregivers" OR "laypersons" OR "guardian" OR "keeper"))

### ACM Digital Library

Title:(("mHealth" OR "mobile app" OR "smartphone application" OR "mobile health" OR "teletriage" OR "telehealth" OR "telemedicine" OR "informatics" OR "software development" OR "software design" OR "application design" OR "application development") AND ("veterinary" OR "animal" OR "pet" OR "dog" OR "cat" OR "horse" OR "ferret" OR "rabbit" OR "guinea pig" OR "rat" OR "mouse" OR "hamster" OR "hedgehog") AND ("owners" OR "caregivers" OR "laypersons" OR "guardian" OR "keeper")) OR Abstract:(("mHealth" OR "mobile app" OR "smartphone application" OR "mobile health" OR "teletriage" OR "telehealth" OR "telemedicine" OR "informatics" OR "software development" OR "software design" OR "application design" OR "application development") AND ("veterinary" OR "animal" OR "pet" OR "dog" OR "cat" OR "horse" OR "ferret" OR "rabbit" OR "guinea pig" OR "rat" OR "mouse" OR "hamster" OR "hedgehog") AND ("owners" OR "caregivers" OR "laypersons" OR "guardian" OR "keeper")) OR Keyword:(("mHealth" OR "mobile app" OR "smartphone application" OR "mobile health" OR "teletriage" OR "telehealth" OR "telemedicine" OR "informatics" OR "software development" OR "software design" OR "application design" OR "application development") AND ("veterinary" OR "animal" OR "pet" OR "dog" OR "cat" OR "horse" OR "ferret" OR "rabbit" OR "guinea pig" OR "rat" OR "mouse" OR "hamster" OR "hedgehog") AND ("owners" OR "caregivers" OR "laypersons" OR "guardian" OR "keeper"))

### IEEE Xplore

(„Document Title“:“mHealth” OR „Abstract“:“mHealth” OR „Author Keywords“:“mHealth” OR „Document Title“:„mobile app“ OR „Abstract“:„mobile app“ OR „Author Keywords“:„mobile app“ OR „Document Title“:„smartphone application“ OR „Abstract“:„smartphone application“ OR „Author Keywords“:„smartphone application“ OR „Document Title“:„mobile health“ OR „Abstract“:„mobile health“ OR „Author Keywords“:„mobile health“ OR „Document Title“:„teletriage” OR Abstract“:„teletriage” OR „Author Keywords“:„teletriage” OR „Document Title“:„telehealth” OR „Abstract“:„telehealth” OR „Author Keywords“:„telehealth” OR „Document Title“:„telemedicine” OR „Abstract“:„telemedicine” OR „Author Keywords“:„telemedicine” OR „Document Title“:„informatics“ OR „Abstract“:„informatics“ OR „Author Keywords“:„informatics“ OR „Document Title“:„software development“ OR „Abstract“:„software development“ OR „Author Keywords“:„software development“ OR „Document Title“:„software design” OR „Abstract“:„software design” OR „Author Keywords“:„software design” OR „Document Title“:„application design” OR „Abstract“:„application design” OR „Author Keywords“:„application design” OR „Document Title“:„application development” OR „Abstract“:„application development” OR „Author Keywords“:„application development”) AND („Document Title“:“veterinary” OR „Abstract“:“veterinary” OR „Author Keywords“:“veterinary” OR „Document Title“:„animal” OR „Abstract“:„animal” OR „Author Keywords“:„animal” OR „Document Title“:„pet“ OR „Abstract“:„pet“ OR „Author Keywords“:„pet“ OR „Document Title“:„dog“ OR „Abstract“:„dog“ OR „Author Keywords“:„dog“ OR „Document Title“:„cat“ OR „Abstract“:„cat“ OR „Author Keywords“:„cat“ OR „Document Title“:„horse“ OR „Abstract“:„horse“ OR „Author Keywords“:„horse“ OR „Document Title“:„ferret” OR „Abstract“:„ferret” OR „Author Keywords“:„ferret” OR „Document Title“:„rabbit” OR „Abstract“:„rabbit” OR „Author Keywords“:„rabbit” OR „Document Title“:„guinea pig“ OR „Abstract“:„guinea pig“ OR „Author Keywords“:„guinea pig“ OR „Document Title“:„rat“ OR „Abstract“:„rat“ OR „Author Keywords“:„rat“ OR „Document Title“:„mouse“ OR „Abstract“:„mouse“ OR „Author Keywords“:„mouse“ OR „Document Title“:„hamster“ OR „Abstract“:„hamster“ OR „Author Keywords“:„hamster“ OR „Document Title“:„hedgehog” OR „Abstract“:„hedgehog” OR „Author Keywords“:„hedgehog”) AND („Document Title“:“owners” OR „Abstract“:“owners” OR „Author Keywords“:“owners” OR „Document Title“:„caregivers” OR „Abstract“:„caregivers” OR „Author Keywords“:„caregivers” OR „Document Title“:„laypersons” OR „Abstract“:„laypersons” OR „Author Keywords“:„laypersons” OR „Document Title“:„guardian” OR „Abstract“:„guardian” OR „Author Keywords“:„guardian” OR „Document Title“:„keeper” OR „Abstract“:„keeper” OR „Author Keywords“:„keeper”)

### EBSCO Host

For TI (Title):

(("mHealth" OR "mobile app" OR "smartphone application" OR "mobile health" OR "teletriage" OR "telehealth" OR "telemedicine" OR "informatics" OR "software development" OR "software design" OR "application design" OR "application development") AND ("veterinary" OR "animal" OR "pet" OR "dog" OR "cat" OR "horse" OR "ferret" OR "rabbit" OR "guinea pig" OR "rat" OR "mouse" OR "hamster" OR "hedgehog") AND ("owners" OR "caregivers" OR "laypersons" OR "guardian" OR "keeper"))

For AB (Abstract):

(("mHealth" OR "mobile app" OR "smartphone application" OR "mobile health" OR "teletriage" OR "telehealth" OR "telemedicine" OR "informatics" OR "software development" OR "software design" OR "application design" OR "application development") AND ("veterinary" OR "animal" OR "pet" OR "dog" OR "cat" OR "horse" OR "ferret" OR "rabbit" OR "guinea pig" OR "rat" OR "mouse" OR "hamster" OR "hedgehog") AND ("owners" OR "caregivers" OR "laypersons" OR "guardian" OR "keeper"))

For SU (Keywords):

(("mHealth" OR "mobile app" OR "smartphone application" OR "mobile health" OR "teletriage" OR "telehealth" OR "telemedicine" OR "informatics" OR "software development" OR "software design" OR "application design" OR "application development") AND ("veterinary" OR "animal" OR "pet" OR "dog" OR "cat" OR "horse" OR "ferret" OR "rabbit" OR "guinea pig" OR "rat" OR "mouse" OR "hamster" OR "hedgehog") AND ("owners" OR "caregivers" OR "laypersons" OR "guardian" OR "keeper"))

### Web of Science

((TI=(“mHealth”) OR AB=(“mHealth”)) OR (TI=(„mobile app“) OR AB=(„mobile app“)) OR (TI=(„smartphone application“) OR AB=(„smartphone application“)) OR (TI=(„mobile health“) OR AB=(„mobile health“)) OR (TI=(„teletriage”) OR AB=(„teletriage”)) OR (TI=(„telehealth”) OR AB=(„telehealth”)) OR (TI=(„telemedicine”) OR AB=(„telemedicine”)) OR (TI=(„informatics“) OR AB=(„informatics“)) OR (TI=(„software development“) OR AB=(„software development“)) OR (TI=(„software design”) OR AB=(„software design”)) OR (TI=(„application design”) OR AB=(„application design”)) OR (TI=(„application development”) OR AB=(„application development”))) AND ((TI=(“veterinary”) OR AB=(“veterinary”)) OR (TI=(„animal”) OR AB=(„animal”)) OR (TI=(„pet“) OR AB=(„pet“)) OR (TI=(„dog“) OR AB=(„dog“)) OR (TI=(„cat“) OR AB=(„cat“)) OR (TI=(„horse“) OR AB=(„horse“)) OR (TI=(„ferret”) OR AB=(„ferret”)) OR (TI=(„rabbit”) OR AB=(„rabbit”)) OR (TI=(„guinea pig“) OR AB=(„guinea pig“)) OR (TI=(„rat“) OR AB=(„rat“)) OR (TI=(„mouse“) OR AB=(„mouse“)) OR (TI=(„hamster“) OR AB=(„hamster“)) OR (TI=(„hedgehog”) OR AB=(„hedgehog”))) AND ((TI=(“owners”) OR AB=(“owners”)) OR (TI=(„caregivers”) OR AB=(„caregivers”)) OR (TI=(„laypersons”) OR AB=(„laypersons”)) OR (TI=(„guardian”) OR AB=(„guardian”)) OR (TI=(„keeper”) OR AB=(„keeper”)))

### PubMed

((“mHealth“[Title/Abstract]) OR („mobile app“[Title/Abstract]) OR („smartphone application“[Title/Abstract]) OR („mobile health“[Title/Abstract]) OR („teletriage”[Title/Abstract]) OR („telehealth”[Title/Abstract]) OR („telemedicine”[Title/Abstract]) OR („informatics“[Title/Abstract]) OR („software development“[Title/Abstract]) OR („software design“[Title/Abstract]) OR („application design”[Title/Abstract]) OR („application development“[Title/Abstract])) AND ((“veterinary”[Title/Abstract]) OR („animal”[Title/Abstract]) OR („pet“[Title/Abstract]) OR („dog“[Title/Abstract]) OR („cat“[Title/Abstract]) OR („horse“[Title/Abstract]) OR („ferret”[Title/Abstract]) OR („rabbit”[Title/Abstract]) OR („guinea pig“[Title/Abstract]) OR („rat“[Title/Abstract]) OR („mouse“[Title/Abstract]) OR („hamster“[Title/Abstract]) OR („hedgehog”[Title/Abstract])) AND ((“owners”[Title/Abstract]) OR („caregivers”[Title/Abstract]) OR („laypersons”[Title/Abstract]) OR („guardian”[Title/Abstract]) OR („keeper”[Title/Abstract]))

### CAB Direct

((title:(“mHealth”) OR ab:(“mHealth”)) OR (title:(„mobile app“) OR ab:(„mobile app“)) OR (title:(„smartphone application“) OR ab:(„smartphone application“)) OR (title:(„mobile health“) OR ab:(„mobile health“)) OR (title:(„teletriage”) OR ab:(„teletriage”)) OR (title:(„telehealth”) OR ab:(„telehealth”)) OR (title:(„telemedicine”) OR ab:(„telemedicine”)) OR (title:(„informatics“) OR ab:(„informatics“)) OR (title:(„software development“) OR ab:(„software development“)) OR (title:(„software design”) OR ab:(„software design”)) OR (title:(„application design”) OR ab:(„application design”)) OR (title:(„application development”) OR ab:(„application development”))) AND ((title:(“veterinary”) OR ab:(“veterinary”)) OR (title:(„animal”) OR ab:(„animal”)) OR (title:(„pet“) OR ab:(„pet“)) OR (title:(„dog“) OR ab:(„dog“)) OR (title:(„cat“) OR ab:(„cat“)) OR (title:(„horse“) OR ab:(„horse“)) OR (title:(„ferret”) OR ab:(„ferret”)) OR (title:(„rabbit”) OR ab:(„rabbit”)) OR (title:(„guinea pig“) OR ab:(„guinea pig“)) OR (title:(„rat“) OR ab:(„rat“)) OR (title:(„mouse“) OR ab:(„mouse“)) OR (title:(„hamster“) OR ab:(„hamster“)) OR (title:(„hedgehog”) OR ab:(„hedgehog”))) AND ((title:(“owners”) OR ab:(“owners”)) OR (title:(„caregivers”) OR ab:(„caregivers”)) OR (title:(„laypersons”) OR ab:(„laypersons”)) OR (title:(„guardian”) OR ab:(„guardian”)) OR (title:(„keeper”) OR ab:(„keeper”)))
